# Prevalence and determinants of fever, ARI and diarrhea among children aged 6–59 months in Bangladesh

**DOI:** 10.1186/s12887-022-03166-9

**Published:** 2022-03-05

**Authors:** Azizur Rahman, Md. Moyazzem Hossain

**Affiliations:** 1 School of Computing, Mathematics and Engineering, Charles Sturt University, Wagga Wagga, NSW 2678 Australia; 2 Department of Statistics, Jahangirnagar University, Dhaka, 1342 Bangladesh

**Keywords:** Childhood illness, Demographic and socio-economic, Community and health, Risk factors, Bangladesh

## Abstract

**Background:**

Although efforts have been made by the international community to improve childhood health, risk factors linked with the healthiness of preschool-age children in low and middle-income countries (LMICs) are very diverse. Therefore, this paper examines the prevalence and determinants of fever, acute respiratory infection and diarrhea of preschool children in Bangladesh.

**Methods:**

A sample of 8,421 children from the latest country representative BDHS-2017–18 survey was analyzed by utilizing both the bivariate and multivariate techniques.

**Results:**

The results revealed that about 4.7, 33.1, and 35.8% of the children aged under 5 years had suffered from diarrhea, fever and ARI respectively during the 2 weeks preceding the date of the survey. Demographic, socio-economic, and community and health characteristics likely to play an important role in suffering under-five children from diarrhea, fever, and ARI in Bangladesh. The child’s age of 13–24 months, delivery by cesarean section, unsafe drinking water, unhygienic toilet facility, low level of family wealth index and parental education, a higher number of living children in the household, rural residency and regional difference were all found to be most crucial determinants of the occurrences of fever, ARI and diarrhea.

**Conclusion:**

Interventions should focus on improving these significant demographic, socioeconomic, and community and health risk factors. A special attention is necessary to the people who live in rural areas and geospatially disadvantaged regions.

## Background

The World Health Organization (WHO) describes health as a state of complete physical, mental and social well-being and not merely the absence of disease or infirmity [1]. Health improvement is an important indicator of the development of any country. The overall health condition of the citizens of Bangladesh is far beneath any acceptable standard [2]. This is especially true for children as well as mothers, who have the poorest health statistics. Although efforts have been made by the international community to improve childhood health, risk factors linked with the healthiness of preschool-age children in low and middle-income countries (LMICs) are very diverse [3]. Globally, around 40 and 60% of children receive satisfactory healthcare regarding diarrhea and acute respiratory infection (ARI) symptoms, respectively, however, diarrhea and ARI is the prominent killer of under-5 mortality still now [4, 5]. ARI, fever, and diarrhea are prominent causes of the burden of disease along with mortality globally, mostly in developing countries [6, 7]. The risks of ARI were noticed to be different by age of children, i.e., younger children suffering more than their older counterparts [8–13]. Preference for better food, healthcare, and treatment facilities can cause the health condition and morbidity of children [6, 7, 14–16]. The membership status of mothers in microcredit organizations has a positive effect on the health condition as well as morbidity of their children [17, 18].

A study suggests that more than 20 percent of under-five children were suffering from cold and fever and 16.69 percent  were sufferings from diarrhea in Bangladesh [19]. Childhood fever is also the most common clinical symptom found in children under 5 years of age and treated as a measure of the public health burden of the disease [20, 21], and in low- and middle-income countries (LMICs), viral infections are the most common cause of fever in children under the age of five [22–24]. In low- and middle-income countries, children under the age of five had 2–9 febrile episodes per year [25]. Fever has increased pediatric consultations, and overtreatment could have a major impact on the economy in low-middle-income and high-income countries [26]. In Odisha, India, a study was conducted to determine the cost of treating febrile illness in children under the age of five, including both direct and indirect costs [24].

The incidence of diarrhea varied according to the categories of toilet facilities operated by households in developing countries [13, 27]. The usage of an improved and safe water source and superior sanitation had a substantial impact on the prevalence of childhood diarrhea in Bangladesh [28, 29] and Ethiopia [30]. The crowding index, as calculated by the number of persons living in a room, also revealed an association with exposure to diarrhea [31]. Children with poor nutritional status is another factor correlated with increased severity of common infectious diseases, as well as death among children with nutritional deficiency is almost always a consequence of these common illnesses [32, 33]. The underlying correlates of childhood health include income poverty, which is concomitant with household food uncertainty. Water, sanitation, and health facilities represent the infection environment that children are exposed to and increase the likelihood of suffering from diseases [34]. Illness in early childhood has a severe effect on children’s health and development, and diarrhea along with acute respiratory infections are the cause of two-thirds of all deaths of children [35].

Like many other developing countries, Bangladeshi children aged under 5 years are also widely suffering from three common diseases such as diarrhea, fever, and ARI in the 2 weeks leading up to the survey. The existing literature suggests that there are several risk factors associated such as age and sex of a child, birth order, place of residence, nutritional status, maternal age, parental education, intimate partner violence, and a better maternal health condition, wealth index, expenditure for health treatment of an individual, access to several media, access to safe water and improved sanitation facilities with the prevalence of diarrhea, ARI, and fever all over the world [3, 6–8, 11–13, 16, 21, 27, 36–51]. However, as far knowledge of the authors, no findings are available for identifying the potential risk factors of diarrhea, ARI, and fever in light of the most recent Bangladesh Demographic and Health Survey (BDHS)-2017–18. Therefore, to fulfill this gap, this study aims to investigate the prevalence of fever, acute respiratory infection, and diarrhea among preschool children in Bangladesh and identify the key determinants of these three common illnesses considering the most recent BDHS-2017–18 dataset.

## Methods and materials

### Data source

This study considers a secondary dataset which is collected from a county representative entitled Bangladesh Demographic and Health Survey (BDHS)-2017–18. The sampling frame of this survey was the list of enumeration areas (EAs) of the 2011 Population and Housing Census of the People’s Republic of Bangladesh. The primary sampling unit of this survey was an EA. The survey used two-stage stratified sampling techniques. In the first stage, a total of 675 EAs were chosen with 227 and 448 EAs from urban and rural areas respectively. However, data was not possible to collect from 3 EAs due to natural disaster. These clusters were in Dhaka (one urban cluster), Rajshahi (one rural cluster), and Rangpur (one rural cluster). In the second stage of sampling, a systematic sample of 30 households selected from each EA. As a result, 20,250 residential households were selected into the four phases. Among the 20,376 ever-married women age 15–49 years eligible for interviews, 20,127 were interviewed, yielding a response rate of 99%. The missing observations are discarded from the analysis and the final sample of 8,421 children aged under-5 years was used for the subsequent analysis. The full data set is available from the following link http://dhsprogram.com/data/available-datasets.cfm. Before starting the analysis the authors use a weighted sample to make sure the country representative sample. The details of the sampling procedure and methods of the weighted sample (mathematically adjusted) are available on the report of the Bangladesh Demographic and Health Survey-2017–18 [52].

### Variables

This study aims to measure the prevalence of diarrhea, fever, and ARI in the 2 weeks of under-5 years’ old children in Bangladesh. The following question was asked to the mother of the child “Did your child suffered from diarrhea, fever, and ARI in the last 2 weeks?” to assessing the prevalence of the disease. The outcome of this question considered as the dependent variable, which is a dichotomous variable indicating whether a child is suffered from the mentioned disease (denoted by “Yes” and coded as “1”) or not (denoted by “No” and coded as “0”).

Although, these three diseases are infectious they have distinct attributes in terms of source of infections and impact on health. For example, the main source of diarrhea could be related to hygienic factors such as washing hand, type of latrine, poor quality of water and food consumption, etc. Typically, children who are suffering from diarrhea cannot consume adequate food because of dehydration as well as cultural factors like mother’s believe about fasting may help to cure from diarrhea. These could lead to the malnutrition in long run as children are suffering from insufficient food intakes. Moreover, supplementary feeding practice can lead to bacterial contamination for poor sterilization, and the frequently taking of plain water or sugar water can be a cause of diarrhea [14]. Besides, fever could be due to mosquito bite, environmental factors like high temperature and monsoon weather, while ARI could be due to coughing, poor air quality, cooking materials used, housing conditions, etc. Both of them impacts on children health adversely in different ways. Additionally, the BDHS survey consider these diseases separately. As a result, this study considers these comorbidity factors of diarrhea, fever and ARI individually. Moreover, the socio-economic and demographic characteristics like age of the child (in months), child’s sex, region/division, religion, number of children, mother’s age, mother’s education, father’s education, wealth index, and so on included in the subsequent analysis as a set of covariates. It is mentioned that the selection of variables was not arbitrary; rather, it was influenced by the availability in the BDHS dataset, existing literature [7, 10, 42–44, 46, 49–52], and self-efficacy. The list of variables with their respective definition and value labels are presented in Appendix.

### Statistical methods

Statistical analyses were performed using SPSS version 24 (SPSS Inc, Chicago, IL). The bivariate analyses using cross-tabulations were also performed separately for three diseases to obtain the association between the occurrence of disease of the children and various categories of the selected variables which are used in multivariate analysis and the significant determinants are explored by the Pearson’s Chi-square *(χ*^*2*^*)* test. The logistic regressions for multivariable analyses was carried out to determining the impact of the explanatory variables on the likelihood of the occurrence of the childhood illness considered in this study. Appropriate multivariate analysis was necessary since the study aimed to find out the significant risk factors of fever, ARI and diarrhea of children. We used three binary logistic regression (adjusted) models for three diseases separately without no confounding factor.

The logistic regression model is expressed as,

$$\mathrm{Pr}\left({Y}_{i}=1\right)=\frac{\mathrm{exp}\left({X}_{i}\beta \right)}{1+\mathrm{exp}\left({X}_{i}\beta \right)}$$

where, *Y*_*i*_ is a binary variable that takes a value of ‘1’ if the respondent has disease and ‘0’ otherwise, *X*_*i*_ is a vector of independent variables and *β* is a vector of unknown parameters.

The estimated form of regression is defined as,

$$\mathrm{In}\left[\frac{{\widehat{P}}_{i}}{1-{\widehat{P}}_{i}}\right]={\widehat{\beta }}_{0}+{\widehat{\beta }}_{1}{X}_{1}+\cdots +{\widehat{\beta }}_{k}{X}_{k}$$

The odds ratio (OR) in favor of *Y*_*i*_ =1 was computed for *X*_1,_*X*_2,…_,*X*_*k*_ to indicate how many times the group of interest is more likely to be associated with the disease of child compared to the reference group. A detailed history of the logistic regression is presented by Cramer in 2002 [53].

## Results

**Fig. 1:**
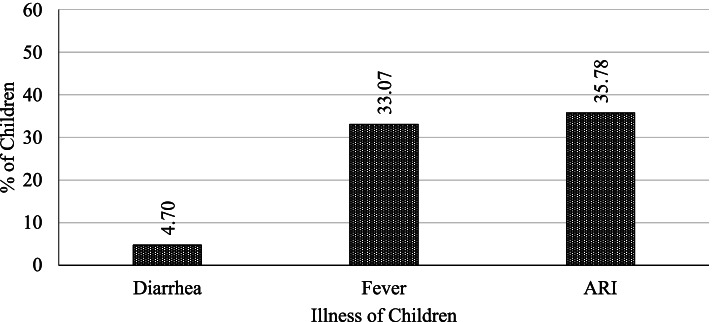
Estimated distribution of child illness in Bangladesh, 2017–18 (*n* = 8,421)

This section provides empirical results and a relevant discussion. All statistical analyses are based on the three common illness variables such as diarrhea, fever, and ARI in the 2 weeks leading up to the survey and selected socio-economic, demographic, and community variables. Figure 1 depicts the percentage estimates of 8,421 study children with their illness status for diarrhea, fever, and ARI. Data demonstrate that about 4.7, 33.07, and 35.78% of the children aged under 5 years had suffered from diarrhea, fever, and ARI respectively. Although the percentage of children who suffered from diarrhea was fairly small, the other two diseases were highly prevalent. Childhood chronic malnutrition, prenatal care, breastfeeding practices, mother education, access to health facilities and mass media communication, vaccine coverage, and household wealth may all be significant contributors to the higher prevalence of fever and ARI.

The distribution of children aged under 5 years who suffered diarrhea, fever, and ARI with selected demographic, socio-economic, and community and health characteristics is presented in Table 1. The results for ARI demonstrate that male children suffer more than their counterparts. Approximately 38% of children aged less than 12 months suffered from ARI, however, the rate for children aged 25 months and above was 33.63% (see Table 1). Both sex and age of the child are highly significant with the prevalence of ARI (*p*-value < 0.001). Mothers whose age at first birth was less than 18 years, had children who were more affected by ARI than the children of mothers whose age at first birth was more than 18 years. Moreover, children who were not breastfeeding were less likely to be suffering from ARI than those who were breastfeeding. The prevalence of ARI is more among the child whose mother’s BMI is categorized as thin compared to normal and overweight mothers. The chance of getting affected by ARI is slightly high for children born by cesarean section than for those who were born by normal procedure. About 33.86% of children who suffered ARI have a modern toilet facility, whereas 36.61 and 35.81% of ARI sufferers use pit or open toilet facilities respectively. The percentage of children living in the poorest wealth index family who suffered ARI was 37.22%, which is higher than the rate (33.46%) for children living in the richest wealth index family. In addition, the prevalence of ARI in preschool children decreases with increasing levels of education of fathers’ education, however, there is an adverse scenario for mother’s education. Children delivered within a health facility (i.e. a hospital) have a slightly higher rate of occurrence of ARI compared to children who were delivered at home. Households with more than two living children have a higher prevalence of ARI compared to households with four or more children. Muslim children were slightly more affected by ARI than non-Muslim children. The prevalence of ARI also varies with geospatial factors. About 36.16% of children from rural areas suffered from ARI, whereas in urban areas the rate was 34.76%. Furthermore, the highest rate of children suffering ARI was 41.92% in the Rangpur division, followed by the Rajshahi division (41.15%), while the lowest rate observed was in the Dhaka division (32.21%) (Table 1).

**Table 1:** Demographic, socio-economic and community and health characteristics of studied children aged 0–59 months and their percentage distribution by three illness (i.e. diarrhea, fever and ARI) in Bangladesh, 2017–18 (*n* = 8,421)

Selected variables	Categories	Diarrhea (%)		*p*-value	Fever (%)		*p*-value	ARI (%)		*p*-value
		**No**	**Yes**		**No**	**Yes**		**No**	**Yes**	
**Demographic**										
Sex of child	Male^a^	94.94	5.06	0.09	65.77	34.23	0.02	62.36	37.64	< 0.001
	Female	95.68	4.32		68.20	31.80		66.25	33.75	
Age of child (in months)	< 12^a^	94.12	5.88	< 0.001	63.44	36.56	< 0.001	62.08	37.92	< 0.001
	13–24	91.21	8.79		60.73	39.27		59.67	40.33	
	25 +	97.19	2.81		70.33	29.67		66.37	33.63	
Currently breastfeeding	No^a^	97.30	2.70	< 0.001	69.61	30.39	< 0.001	66.07	33.93	< 0.001
	Yes	93.89	6.11		65.05	34.95		62.93	37.07	
Age of mother at 1st birth (Years)	12-17^a^	95.38	4.62	0.33	65.44	34.56	0.03	63.40	36.60	0.24
	18–25	95.12	4.88		67.92	32.08		64.66	35.34	
	26 +	96.96	3.04		70.17	29.83		67.57	32.43	
Mother's BMI	Thin^a^	94.07	5.93	0.11	65.39	34.61	0.05	61.81	38.19	0.24
	Normal	95.43	4.57		66.14	33.86		64.39	35.61	
	Overweight	95.56	4.44		68.79	31.21		64.46	35.54	
**Socio-economic**										
Father’s education	No^a^	96.28	3.72	0.03	67.83	32.17	0.77	64.78	35.22	0.58
	Primary	94.51	5.49		66.94	33.06		63.51	36.49	
	Secondary +	95.46	4.54		66.72	33.28		64.63	35.37	
Mother’s education	No^a^	93.78	6.22	0.18	69.89	30.11	0.25	70.59	29.41	< 0.001
	Primary	95.47	4.53		66.33	33.67		64.77	35.23	
	Secondary +	95.41	4.59		66.87	33.13		63.26	36.74	
Wealth index	Poorest^a^	95.09	4.91	0.01	65.81	34.19	< 0.001	62.78	37.22	0.21
	Poorer	95.38	4.62		66.73	33.27		63.57	36.43	
	Middle	94.08	5.92		65.47	34.53		64.02	35.98	
	Richer	96.69	3.31		64.87	35.13		64.36	35.64	
	Richest	95.19	4.81		71.89	28.11		66.54	33.46	
**Community and health**										
Place of delivery	With Health Facility^a^	93.30	6.70	0.53	64.86	35.14	0.06	60.23	39.77	0.07
	Respondent's Home	93.73	6.27		62.37	37.63		62.68	37.32	
Delivery by caesarean section	No^a^	93.51	6.49	0.93	63.31	36.69	0.56	62.14	37.86	0.08
	Yes	93.57	6.43		64.14	35.86		60.16	39.84	
No. of living children	1^a^	94.79	5.21	0.04	67.01	32.99	0.30	61.58	38.42	< 0.001
	2–3	95.32	4.68		67.34	32.66		65.34	34.66	
	4 or more	96.81	3.19		64.69	35.31		66.89	33.11	
Religion	Muslim^a^	95.13	4.87	0.02	66.45	33.55	< 0.001	64.14	35.86	0.59
	Non-Muslim	97.17	2.83		72.47	27.53		65.18	34.82	
Place of residence	Urban^a^	95.58	4.42	0.47	69.05	30.95	0.01	65.24	34.76	0.23
	Rural	95.21	4.79		66.14	33.86		63.84	36.16	
Source of drinking water	Safe^a^	95.12	4.88	0.04	66.53	33.47	0.04	63.94	36.06	0.18
	Unsafe	96.42	3.58		69.45	30.55		65.96	34.04	
Type of toilet facility	Modern^a^	95.49	4.51	0.23	68.89	31.11	0.04	66.14	33.86	0.08
	Pit Latrine	95.02	4.98		65.95	34.05		63.39	36.61	
	Others	96.15	3.85		67.45	32.55		64.19	35.81	
Received BCG	No^a^	98.88	1.12	< 0.001	79.61	20.39	< 0.001	80.73	19.27	< 0.001
	Yes	93.14	6.86		62.43	37.57		60.03	39.97	
Received DPT	No^a^	96.57	3.43	< 0.001	71.20	28.80	< 0.001	69.66	30.34	< 0.001
	Yes	92.81	7.19		61.82	38.18		59.52	40.48	
Division	Barisal^a^	93.53	6.47	0.11	61.64	38.36	0.01	62.28	37.72	< 0.001
	Chittagong	94.95	5.05		67.52	32.48		66.95	33.05	
	Dhaka	96.18	3.82		69.26	30.74		67.79	32.21	
	Khulna	96.12	3.88		68.73	31.27		63.95	36.05	
	Mymensingh	94.63	5.37		66.67	33.33		62.43	37.57	
	Rajshahi	94.27	5.73		65.40	34.60		58.85	41.15	
	Rangpur	95.59	4.41		63.73	36.27		58.08	41.92	
	Sylhet	95.45	4.55		66.18	33.82		65.01	34.99	

^a^Reference category in the multivariate analysis

[Note: *p*-value < 0.1 for at least one disease is considered in identifying the significant determinants.]

It is seen that male child and whose age is between 13 to 24 months suffer more from fever. Results of the distribution of study children who suffered from fever show that the prevalence of fever decreases with increasing the age of the mother at the first childbirth. Children who were currently breastfeeding status had a little bit higher chance of suffering from fever than their counterparts. Though the reality is that the breastfeeding practice had a chance to lessen the prevalence of childhood illness, however, among children who take additional foods and fluids that are potentially contaminated and may increase the likelihood of suffering from several diseases. In addition, children from mothers who do not have access to safe drinking water, modern sanitation facilities, or who live in poor conditions are more likely to suffer from the various diseases associated with fever, because their mother acts as a carrier of different microorganisms that may be imported into their body. The percentage of children with fever decreases as the household wealth index increases. Children who are born by cesarean section and without health facilities have a less likelihood of experiencing fever. Additionally, access to a modern toilet facility decreases the likelihood of fever for a child. Households with more than four children had a higher rate of children who suffered from fever compared to households with only one or two children. Furthermore, children from rural areas were slightly more affected by fever than children from urban areas. The highest percentage of children with fever was observed in the Barisal division (38.36%) and the lowest was in the Dhaka division (30.74%) (Table 1).

The results reveal that diarrhea is more prevalent for children aged less than 25 months compared to age groups of more than 25 months. A child who is currently breastfeeding had higher exposure to diarrhea. Among the three categories of the age of the mother at first birth, the occurrence of diarrhea among children is more in cases where the mother’s age at first birth was between 18 to 25 years. The prevalence of diarrhea in children who were delivered by cesarean section is almost similar to their counterparts, however, have access to a modern toilet facility and a rich wealth index is fairly low. For example, about 3.31% of children from wealthy families were affected by diarrhea, while 5.92 and 4.62% of children suffered from diarrhea in middle and poor families respectively. The children whose mothers have an education level secondary and above are less affected by diarrhea than those of parents with no education. Moreover, children from rural areas are slightly more affected by diarrhea (4.79%) than those from urban areas (4.42%). Muslim children have more exposure to diarrhea than their non-Muslim counterparts. The highest rate of occurrence of diarrhea was observed in the Barisal division (6.47%), followed by the Rajshahi division (5.73%), and the lowest rate was observed in the Dhaka division (3.82%) (Table 1).

The results of logistic regression analysis on diarrhea, fever, and ARI by selected demographic, socio-economic and community and health characteristics are presented in Table 2. The findings reveal that sex and age of child, current breastfeeding status, method of delivery, sources of drinking water, toilet facility, wealth index, parental educational status, number of children, childhood residence, a child received BCG and DPT vaccination and region have somewhat significant effects on the prevalence of diarrhea, fever, and ARI. In particular, children aged 13–24 months and 25 months and above are 1.31 times more likely and 0.65 times less chance to be exposed to diarrhea respectively than children aged less than 12 months. A child whose mother’s age at 1^st^ birth is 26 years and more is about 50% less likely to be infected in diarrhea than a child whose mother’s age at birth is less than 18 years. Children from rich families had nearly 28% less chance of suffering from diarrhea respectively than children from the poorest families. Children whose mother’s educational levels were primary and secondary or higher had approximately around 50% less chance of being affected by diarrhea than children with a mother who had no formal education. Children residing in rural areas had a little bit more likelihood of being exposed to diarrhea than their urban counterparts. The results demonstrate that children from the Chittagong, Dhaka, Khulna, Mymensing, Rajshahi, Rangpur and Sylhet divisions had a lower risk of experiencing diarrhea than children from the Barisal division. Moreover, the findings presented in Table 2 also depict that children aged more than 12 months were more likely to suffer from fever than children aged less than 12 months. Female children are slightly less likely to suffer from fever than male children. Currently breastfeeding children had about 9% less chance of getting fever compared to their counterparts. The results of logistic regression for ARI of the selected characteristics reveal that sex, age of the child, place of delivery, sources of drinking water, toilet facility, mother’s educational status, having the BCG and DPT vaccination, place of residence and wealth index were major differentials of ARI for children in Bangladesh. Furthermore, the results of Omnibus Tests of Model Coefficients and Hosmer and Lemeshow test for each model indicate that the fitted models are good (Table 2).

**Table 2:** Regression coefficients and odds ratios for prevalence of diarrhea, fever and ARI by selected demographic, socio-economic and community and health characteristics of children in Bangladesh, 2017–18

**Selected variables**	**Categories**	**Diarrhea**^**b**^** (%)**	**Fever**^**b**^** (%)**	**ARI**^**b**^** (%)**
		**Adjusted OR (95% CI)**	**Adjusted OR (95% CI)**	**Adjusted OR (95% CI)**
**Demographic**				
Sex	Male^a^	-	-	-
	Female	0.95 (0.76,1.2)**	0.92 (0.82,0.98)*	0.79 (0.70,0.89)***
Age of child (in months)	< 12^a^	-	-	-
	13–24	1.11 (0.84,1.46)	0.86 (0.74,1.00)*	0.89 (0.77,1.04)**
	25 +	0.65 (0.46,0.93)**	0.66 (0.55,0.78)***	0.80 (0.67,0.95)**
Currently breastfeeding	No^a^	-	-	-
	Yes	1.44 (0.96,2.15)	0.92 (0.76,0.97)*	0.99 (0.83,1.21)
Age of mother at 1st birth (Years)	12-17^a^	-	-	-
	18–25	1.07 (0.84,1.37)	0.90 (0.80,1.02)*	0.91 (0.8,1.03)
	26 +	0.50 (0.21,1.17)**	0.8 (0.57,1.13)	0.79 (0.57,1.11)
Mother's BMI	Thin^a^	-	-	-
	Normal	0.82 (0.60,1.12)	0.94 (0.79,1.11)	0.93 (0.79,1.10)
	Overweight	1.01 (0.69,1.48)	0.86 (0.70,1.06)	0.97 (0.79,1.19)
**Socio-economic**				
Father’s education	No^a^	-	-	-
	Primary	1.32 (0.89,1.96)	1.04 (0.86,1.27)	1.02 (0.84,1.24)
	Secondary +	1.17 (0.76,1.78)	1.08 (0.87,1.32)	0.98 (0.8,1.21)
Mother’s education	No^a^	-	-	-
	Primary	0.52 (0.33,0.83)**	1.02 (0.78,1.32)	0.98 (0.76,1.28)
	Secondary +	0.48 (0.31,0.77)***	1.06 (0.81,1.38)	1.09 (0.83,1.42)**
Wealth index	Poorest^a^	-	-	-
	Poorer	0.96 (0.67,1.39)	0.89 (0.74,1.08)**	0.89 (0.74,1.07)
	Middle	1.31 (0.90,1.90)**	1.03 (0.85,1.26)***	0.85 (0.7,1.04)
	Richer	0.79 (0.50,1.23)**	1.01 (0.82,1.26)	0.87 (0.7,1.08)**
	Richest	1.07 (0.63,1.82)	0.78 (0.60,0.83)*	0.87 (0.67,1.14)
**Community and health**				
Place of delivery	With health facility^a^	-	-	-
	Respondent's home	0.81 (0.58,1.11)	1.16 (0.98,1.38)	0.92 (0.77,0.98)*
Delivery by caesarean section	No^a^	-	-	-
	Yes	0.92 (0.65,1.29)	1.19 (0.99,1.42)	1.03 (0.86,1.23)
No. of living children	1^a^	-	-	-
	2–3	0.95 (0.74,1.22)	1.13 (0.99,1.29)	0.89 (0.78,0.94)*
	4 or more	0.60 (0.36,0.98)**	1.30 (1.04,1.64)**	1.00 (0.79,1.26)
Religion	Muslim^a^	-	-	-
	Non-Muslim	0.54 (0.32,0.93)**	0.77 (0.61,0.97)**	0.94 (0.76,1.17)
Place of residence	Urban^a^	-	-	-
	Rural	1.01 (0.74,1.37)	1.04 (0.89,1.21)	0.98 (0.84,1.14)
Delivery by caesarean section	No^a^	-	-	-
	Yes	0.92 (0.65,1.29)	1.19 (0.99,1.42)	1.03 (0.86,1.23)
Source of drinking water	Safe^a^	-	-	-
	Unsafe	0.59 (0.29,1.22)**	0.79 (0.67,0.91)*	0.73 (0.53,0.81)*
Type of toilet facility	Modern^a^	-	-	-
	Pit Latrine	0.88 (0.61,1.27)	0.92 (0.76,1.11)	1.09 (0.90,1.31)
	Others	0.90 (0.42,1.93)	1.17 (0.82,1.68)	1.45 (1.01,2.07)**
Received BCG	No^a^	-	-	-
	Yes	3.84 1.33,11.04)**	1.98 (1.45,2.70)***	2.36 (1.72,3.23)***
Received DPT	No^a^	-	-	-
	Yes	1.85 (1.20,2.85)**	1.45 (1.19,1.77)***	1.29 (1.06,1.58)**
Division	Barisal^a^	-	-	-
	Chittagong	0.84 (0.52,1.36)	0.85 (0.65,1.13)	0.95 (0.72,1.25)
	Dhaka	0.56 (0.34,0.93)**	0.80 (0.60,0.95)*	0.94 (0.71,1.25)
	Khulna	0.54 (0.29,0.99)**	0.86 (0.63,1.18)	1.12 (0.81,1.53)
	Mymensingh	0.74 (0.42,1.29)	0.81 (0.59,1.11)**	1.03 (0.76,1.41)
	Rajshahi	0.80 (0.47,1.34)	0.96 (0.72,1.30)	1.4 (1.04,1.88)**
	Rangpur	0.67 (0.39,1.15)	1.03 (0.76,1.39)**	1.27 (0.94,1.71)
	Sylhet	0.82 (0.45,1.47)	1.07 (0.77,1.47)	1.17 (0.85,1.62)**
**Model fitting information**				
Cox & Snell R Square		0.68	0.72	0.76
Omnibus Tests of Model Coefficients		109.35***	123.92***	138.98***
Hosmer and Lemeshow Test		6.98***	6.06***	5.49***

^a^Reference category of each independent variable

^b^Reference category of each dependent variable is ‘no’ i.e. children had not suffered diarrhea, fever or ARI

^*^p < 0.05, **p < 0.01, ***p < 0.001

## Discussion

This study aims to find the potential covariates of the prevalence of diarrhea, fever, and ARI based on the nationally representative 2017–2018 Bangladesh DHS data. The association between the prevalence of the targeted disease with several socio-economic, demographic, and health factors were examined among under-5 children in Bangladesh. The number of diarrheal episodes, ARI symptoms, and fever for the youngest children under 5 years old in the 2 weeks prior to the BDH survey were the outcome variables. The authors created three binary variables, each representing one if a disease is present and zero if it is not. Another study considers these three diseases separately considering the BDHS data from 1993–2014 [54]. Although the percentage of children who suffered from diarrhea was fairly small, the other two diseases were highly prevalent. Childhood chronic malnutrition and it’s determinants such as prenatal care, breastfeeding practices, mother education, access to health facilities and mass media communication, vaccine coverage, and household wealth may all be significant contributors to the higher prevalence in fever and ARI. Despite the fact that diarrhea affected a small number of children, the other two illnesses were extremely common. Childhood chronic malnutrition, prenatal care, breastfeeding habits, mother age and education, birth interval, availability to health facilities and mass media communication, immunization coverage, and household affluence could all have a role in Fever and ARI's increased occurrence [54–60].

Findings reveals that male children are more prone to childhood illness than female children. The probable reason may be that male children, on the other hand, have a predisposition to play outside the home, which exposes them to infected aerosols from the surrounding outside environment [43]. It has been noticed that older children have a lower risk of developing illness investigated in this study. The possible reason is that as children get older, they develop immunity and know how to interact better with their environment, such as avoiding unsanitary areas and eating healthy foods, which indicates a lessening in the occurrence of childhood diseases as they get older [9, 40, 61]. Maternal age and education play a key role in controlling the childhood illness because literacy levels may influence the level of awareness and access to health information [40, 48, 62]. Educated mothers also understand the symptoms of childhood illness that is helpful to lessen the prevalence of neonatal morbidity and mortality in Bangladesh [63, 64]. Moreover, mothers who have low level of schooling, have limited knowledge and practice in terms of childcare, nutrition, health communication and services, maintaining hygienic environment, breastfeeding, and medical complications [54, 65]. In addition, education may raise the mother's understanding of diarrhea transmission and preventative strategies [66]. Surprisingly, breastfeeding status was also not a significant factor for diarrhea and ARI in this study which is consistent with another study [40]. It is believed that safe drinking water and better toilet facilities play a significant role in the occurrence of diarrhea [9, 39, 67]. However, the results of this study indicate that children who do not get access to safe drinking water have less risk of suffering from diarrhea than their counterparts. Surprisingly, an adverse result is found in this study for the children who received BCG and DPT vaccinations and results show that they were highly likely to be exposed to diarrhea respectively than their counterparts though it is not expected.

Children who were born by cesarean section had a greater risk of being affected by fever than children who were born normally. Access to a healthier, modern toilet facility had a significant impact in reducing the risk of getting fever for children aged under five. The modern sanitation and household environment will act to reduce pathogen transmission through fecal contamination [68]. A better family wealth index also had a significant impact on fever incidence, i.e. children from the poorest families have more chance to be affected by fever than children of the richest families [3, 11, 40, 48, 67]. In terms of the economy, household poverty may be a major role in lower living standards, access to high-quality care, and chronic nutritional deficiencies, which leads to a weakened immune system and, as a result, increased disease vulnerability [69, 70]. Children living with more than two siblings had a significantly greater risk of experiencing fever than children living with only one or no siblings. A non-muslim child has less likelihood of suffering from fever than a Muslim child. Children in the Chittagong, Dhaka, Khulna, and Mymensing had less chance of suffering from fever than Barisal division, however, in Rangpur and Sylhet divisions had a little bit more chance to experience fever than children in the Barisal division and a part of these findings is matched with another study [48]. The regional variation may be due to several factors like air pollution, temperature, materials used for cooking, access to healthcare facilities, and so on [71].

In particular, the risk of a child being exposed to ARI was significantly less among female children, after the second year of life, for normally delivered babies, for children having modern latrines, for children living in rural areas and with two or more siblings. These predictors have a potential link with important risk factors such as of fecal contamination of food, drinking water, utensils, and child’s toys [40, 43, 48, 72]. Conversely, the ARI risk was significantly high for children who are residing in the regions that are located in the northwest (i.e. Rajshahi, Rangpur) parts of the country. This is maybe due to the fact that these regions have low poverty rates with much better socioeconomic conditions than the newly-created Barisal division located in the southern and middle parts of the country. This discrepancy could be explained by differences in the socio-demographic, environmental, and behavioral features of households [66].

### Strengths and limitations of this study

The findings of this study are based on the largest and most recent nationally representative surveys of Bangladesh. Several previous studies consider separately the main three childhood diseases, however, three diseases were included in this study. Sometimes children of age under five may suffer from multiple illnesses like ARI and diarrhea is linked with fever or diarrhea is associated with ARI i.e., one is impacted by others, however, in this study we do not consider the mixed illness. The authors will consider the co-occurrence of three common morbidities along with confounding factor in their future research. Moreover, age categories of the children is also play a vital role as young children aged (12- < 24 months) and > 24 months interact with environmental factors. Therefore, the authors think that it would be future research for pointed out the association among different illnesses and their potential covariates keeping the age categories in mind. Along with BCG and DPT vaccination, rotavirus also potential factor for childhood illness, however, we cannot find any relevant information about rotavirus vaccination in the data set. Furthermore, there may be spatial and temporal variation exist among the prevalence of the diseases considered in this study but the authors do not consider it here and it would be a potential topic for further study.

## Conclusions

The study concludes that childhood illness is one of the major public health concerns in Bangladesh as many children aged under 5 years had suffered from diarrhea, fever, and ARI respectively. The age and sex of the child, mother’s educational level, unsafe drinking water, wealth index, a higher number of living children, vaccination status and regional difference are strongly related to occurrences of fever, ARI, and diarrhea. Therefore, interventions should focus on educating parents, and geospatially disadvantaged regions on the importance of immunization practices, having a safe source of drinking water, and family planning to improve mothers’ conditions (e.g., mothers’ age at the first childbirth) and limit the number of children per family. Though the deaths due to diarrhea are declined for taking public–private partnership of the government with SMC, BRAC, and other NGOs to produce more oral rehydration solution (ORS) and make available. However, optimize the coverage of different interventions, promote nutrition and Water, Sanitation and Hygiene (WASH) strategies are needed to continue for lessening the prevalence of childhood illness. Further research should explore the links between these three illnesses and childhood malnutrition with quantile regression approach [62] and the diverse impact of these illnesses on childhood morbidity and mortality to address the urgency for child-survival programs in the population.

## Data Availability

The data set used in this study will be available from the website The DHS Program. In order to gain access to the data files, you have to complete the registration. The data set is available from the following link http://dhsprogram.com/data/available-datasets.cfm.

## Appendix

Table 3

**Table 3:** List of variables with their respective definition and value labels

Category of variables	Variables/description of variables	Value Labels
**Target variables**	Had diarrhea recently?	0 = No; 1 = Yes
	Had fever in last two weeks?	0 = No; 1 = Yes
	Had cough in last two weeks?	0 = No; 1 = Yes
**Covariates**		
**Demographic**	Sex of child	1 = Male; 2 = Female
	Age of child	1 = < 12 months; 2 = 13–24 months; 3 = 25 + months
	Currently breastfeeding status	0 = No/don’t know; 1 = Yes
	Age of mother at 1st birth	1 = 12–17 years; 2 = 18–25 years; 3 = 26 + years
	Mother's BMI	1 = Thin; 2 = Normal; 3 = Overweight
	Husband/partner's education level	0 = No education; 1 = Primary; 2 = Secondary and above
	Mother’s education level	0 = No education; 1 = Primary; 2 = Secondary and above
	Wealth index	1 = Poorest; 2 = Poorer; 3 = Middle; 4 = Richer; 5 = Richest
**Community and health**	Place of delivery	0 = With Health Facility; 1 = Respondent's Home
	No. of living children	1 = 1; 2 = 2–3; 3 = 4 or more
	Religion	1 = Muslim; 2 = Non-muslim
	Type of place of residence?	1 = Urban; 2 = Rural
	Delivery by caesarean section	0 = No; 1 = Yes
	Source of drinking water	0 = Safe; 1 = Unsafe
	Type of toilet facility	1 = Modern; 2 = Pit Latrine; 3 = Others
	Received BCG	0 = No received BCG; 1 = Received BCG
	Received DPT	0 = No received DPT; 1 = Received DPT
	Division	1 = Barisal; 2 = Chittagong; 3 = Dhaka; 4 = Khulna; 5 = Mymensingh; 6 = Rajshahi; 7 = Rangpur; 8 = Sylhet

## Abbreviations

ARI: Acute respiratory infection
BCG: Bacillus calmette–guérin
BDHS: Bangladesh demographic and health survey
DPT: Diphtheria, pertussis and tetanus
LMIC: Low and middle income country
NIPORT: National institute for population, research and training
USAID: United States agency for international development
WHO: World health organization
